# Detection of Crack Locations in Aluminum Alloy Structures Using FBG Sensors

**DOI:** 10.3390/s20020347

**Published:** 2020-01-08

**Authors:** Weifang Zhang, Meng Zhang, Yudong Lan, Yan Zhao, Wei Dai

**Affiliations:** 1 School of Reliability and Systems Engineering, Beihang University, 37 Xueyuan Rd., Haidian Dist., Beijing 100191, China; 08590@buaa.edu.cn (W.Z.); zhangmeng123@buaa.edu.cn (M.Z.); staillyd@buaa.edu.cn (Y.L.); 2 School of Energy and Power Engineering, Beihang University, 37 Xueyuan Rd., Haidian Dist., Beijing 100191, China; zy_buaa@buaa.edu.cn

**Keywords:** FBG sensor, FWHM, spectral difference, crack locations detection, structural health monitoring

## Abstract

This study investigated the reflected spectral deformation mechanism of fiber Bragg grating (FBG) sensors with crack propagation. This analysis was performed based on the simulated FBG response by applying modified-transfer matrix modeling (TMM) with the strain states, which were extracted by the finite element method (FEM) analysis. Experimental data were obtained from FBG sensors bonded in an aluminum alloy structure and subjected to multiple crack lengths, and the strain values were obtained by digital image correlation (DIC) technology. Based on the simulations and the experimental full spectral response, we compared the performance of two damage features: The full width at half maximum (FWHM) and the spectral difference. In addition, results showed that the two features were insensitive to experimental noise and were highly sensitive to the complex strain field caused by crack propagation. Moreover, the damage features changes in the crack propagation process also provided a way for crack position measurement. Ultimately, the 10 mm grating lengths sensors showed better performance to the crack detection with longer sensitivity distance. According to the research in this paper, the crack position was quantitatively determined by evaluating different damage features of the reflected spectrum.

## 1. Introduction

Crack damage usually occurs in aircraft structures. However, it only can be detected by global sensors due to the changing of significant structural properties when the damage is at a high level [1]. Hence, local-level sensors, such as the FBG sensors, are sensitive to small-scale damage and have been successfully used for fatigue crack detection [2]. They also have the advantages of small size, multiplexing ability [3] and strong resistance to external electromagnetic interference [4]. The FBG monitoring techniques obtain periodic responses from sensor arrays, followed by the extracted damage sensitive features analysis, to determine the current state of structural health [5]. Typically, when crack propagation is near the bonded FBGs in a material, the deformation reflected spectra are observed due to the inhomogeneities of both the periods and the effective refractive index, which are caused by strain concentrations, strain gradients, multiple strain components along the FBG axis. Moreover, the spectra can be theoretically predictable with known specific strain states by TMM [6]. However, damage detection based on the presence of deformation FBG reflected spectra under an unknown strain state of an inverse problem will show more difficulty. Hence, extracting information from the simulated and measured FBG reflected spectra is potentially a useful way to monitor the crack state in structures and solve this inverse problem.

In the practical applications, damage sensitive features are required to be sensitive to spectral distortion caused by the uncertain damage state in materials. Additionally, the features are expected to have a low sensitivity to average strain components along the optical fiber, which is dominated by the magnitude of the loading applied to the structure. In addition, the measurements should be sensitive to the nonhomogeneous strain field loading along the optical fiber caused by the presence of damage. Finally, the choice of damage features should consider the central wavelength shifting caused by the temperature fluctuation effect. Consequently, a Bragg wavelength peak shifting is excluded from the damage features because the feature is converted into a measure of the uniform strain along the axis of the grating and the temperature changes [7]. Accordingly, if the damage propagation is close to the sensor, the spectral distortion and sensitivity features extracted from the signal response can be used to detect structural damage. Okabe et al. [8] proposed the spectral bandwidth to measure transverse cracks in cross-ply laminates based on a strong association between the feature and the transverse crack density. Kara Peters et al. [9] showed that the spectral bandwidth and the cross-correlation coefficient presented a high sensitivity to fast-growing strain fields caused by structure cracks and insensitivity to noise. Xin Jin et al. [10] showed that the intensity ratio of primary and secondary peaks was a good measure to detect the extent of damage.

In Section 2 of this paper, two damage features are proposed: FWHM and the spectral difference. The bandwidth was related directly to strain gradients [11]. Additionally, the spectral difference algorithm based on the dynamic time warping (DWT) method overcame the limitations of traditional cross-correlation coefficient definition. It not only considers the phenomenon of reflected spectrum shifting during crack propagation [12] but also ignores the requirement of same comparative signal lengths in traditional definition [13]. Each feature was tested for two cases: First, simulated FBG reflected spectra under a strain state which were extracted by FEM analysis for different crack lengths. These simulation signals were used to evaluate the theoretical sensitivity of each feature to different damage stages. Second, in Section 3, the damage features were extracted from the experimental data of multiple sensors glued to a plate. The experiment environment contained complicated strain fields and realistic test noise, therefore, causing variations of sensor responses. Furthermore, the experimental data were used to determine whether each damage feature could be a reliable indicator to identify the damage state in a realistic environment. The DIC technique was adapted to detect strain field in the experiments. Previous research on fatigue crack propagation using the DIC method were originally developed by using a two-dimensional correlation of the deformed and undeformed gray images of an object [14]. However, the more common ex situ DIC technique that was developed for strain measurements of fatigue crack propagation only considers the changes in the single loading cycle [15] with relatively small covered areas (less than 100 μm) [16]. In this study, full-field measurements during different loading cycles in aluminum alloy are considered to explain the FBG performance. In Section 4, the experiment was carried out, then, the relationship between the FBG spectral distortion and the strain extracted by the DIC technology is discussed. Changes in the damage sensitivity features were used for crack location measurements.

The proposed approach has four key advantages over the previous studies. First, an interpretation of reflected spectrum deformation mechanism with crack propagation were analyzed based on both simulations and experiments. Second, the performance of two potential damage features—FWHM and the spectral difference were proposed and evaluated. Third, the curve of damage features changing with crack propagation presented a significant break point, which corresponds to the moment of crack propagation toward FBG sensors. Fourth, the 10 mm FBG sensor may show superior performance compared to the 5 mm FBG sensor for crack position detection.

## 2. Theoretical Performance

### 2.1. The Extraction of Strain Using Finite Element Modeling

**Figure 1:**
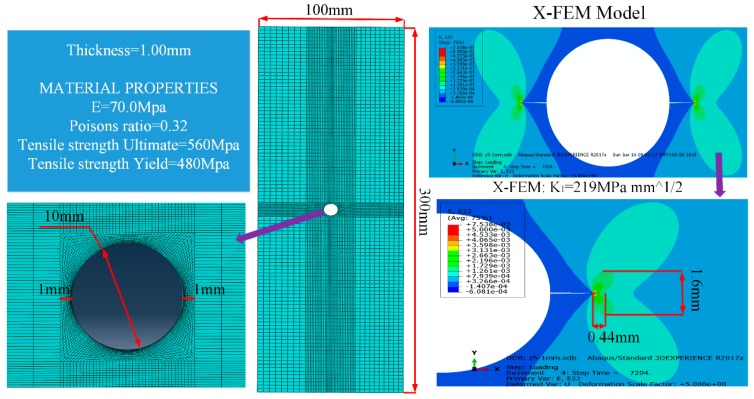
Crack propagation simulation for a 7075-T6 aluminum alloy with FEM method by ABAQUS software.

The specimens for the target system were made of a 7075-T6 aluminum alloy, which is widely used in aircraft and the mechanical peripeties, shown in Figure 1. The specimens with dimensions of 300 × 100 × 1 mm, a 10 mm center hole and a 1 mm through-thickness pre-crack were processed by electric-discharge machining (EMD) to promote fatigue crack initiation.

To stabilize the fatigue crack growth behavior during the loading with force control, the NASGRO software was applied to calculate the reasonable stress magnitude. Rice [17] concluded that the plastic deformation at the fatigue crack tip and the crack growth rate were determined by the variation in the crack tip stress intensity factor (SIF). In this paper, according to the fatigue crack propagation behavior in the NASGRO database, for a constant amplitude fatigue experiment with 1 mm thick 7075-T6 aluminum alloy materials, the stress intensity factor was 224 MPa $\sqrt{mm}$ when the load amplitude was approximately 50 MPa, and the initial fatigue crack growth rate was near 5000 cycles per millimeter. Based on a stress ratio of 0.1, the fatigue load cycling was set to a sinusoidal wave with a maximum of 50 MPa. The applied holding load force on the specimens needs to be constant during spectral data acquisition, and a setting of 70–90% of the maximum force may be perfect considering the effects of crack retardation or crack arrest caused by high holding loads [18]. Therefore, the holding load was set to 40 MPa.

Based on the crack tip strain distributions, which were calculated by the extended finite element method (XFEM), the singular strain field near a crack tip was an important factor adopted to optimize FBG sensor placement. The C3D8R hexahedron-8-node reduced integration elements were used to construct the crack-tip mesh. The refined mesh size around the crack region was 0.05 mm and the remaining mesh size was 1 mm. As a result, the width and the length of the primary plastic zone at the tip of the crack were approximated to be 0.44 and 1.6 mm, respectively, as shown in Figure 1.

**Figure 2:**
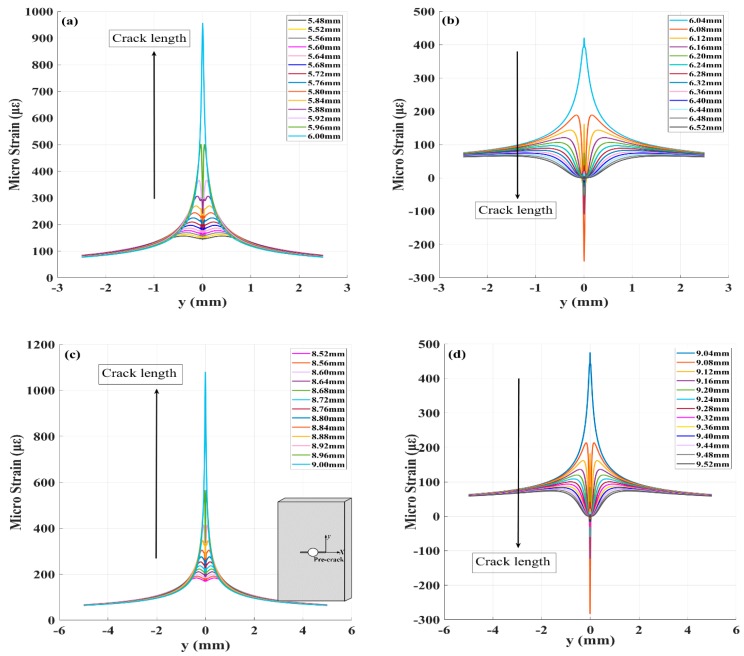
(**a**) The strain along the grating before crack propagation to the FBG1 sensor; (**b**) the strain along the grating after crack propagation across the FBG1 sensor; (**c**) the strain along the grating before crack propagation to the FBG2 sensor; (**d**) the strain along the grating after crack propagation across the FBG2 sensor.

According to previous studies [19], due to the small range of strain inhomogeneity and discontinuity around the crack tip, FBG sensors must be bonded near the crack tip to sense singular stress fields. A 6 mm radial distance from the center hole was selected for placement of the FBG1 sensor (the grating length is 5 mm). The strain along the grating at the FBG1, FBG2 (the grating length is 10 mm), FBG3 (at the same position to FBG2 with 5 mm grating length) sensors under different crack lengths is shown in Figure 2. Additionally, for future strain extractions by the DIC method, the distance between the two sensors should be larger than the crack tip plastic area. The two sensors with different grating lengths and a spacing of 3 mm were bonded at different distances to the crack location to evaluate the robustness of damage features to noise and the damage sensitivity distance of each FBG.

For an ideal crack, in the ε_yy_ plots, the strain filed corresponded with the pre-crack tip, shown in Figure 2. When the FBG sensor was at a distance from the crack tip, it sensed elastic behavior. When a fatigue crack grew under several fatigue loading cycles, the crack path gradually became straight, although the initial crack had a tortuous shape. As crack propagation approached near the FBG sensors, the strains gradually accumulated in the tensile direction with strain concentrations, strain gradients, and multiple strain components along the fiber. A symmetrical plastic zone was expected near the crack tip at both sides of the crack line, and the grating sensing a large quadratic strain. However, for real fatigue cracks, the fiber behind the crack tip senses the residual compressive strains ε_yy_, which exist as a significant portion of the strain composition in the crack wake. According to previous studies on the mechanisms for fatigue crack closure and the mechanisms under plane strain [20], it was reported that the residual strain in the crack wake under plane strain from contraction of the material in the crack propagation direction would help the crack closure.

### 2.2. The Reflected Spectra Simulations

Peters first proposed the modified-TMM methods with fiber under a longitudinally uniform strain field [6]. To study the FBGs reflected spectral subjected to a nonuniform strain field detected by the FEM, the reflection spectra under different strain distributions were simulated by modified-TMM. The T-matrix approach can be viewed as an assembly of piecewise-uniform approaches [21]. Thus, the FBG sensor is a combination of piecewise-uniform sections, each of which is assumed to be a uniform FBG, and the closed-form solution for these sections can be used. The parameters of FBGs are shown in Table 1.

**Figure 3:**
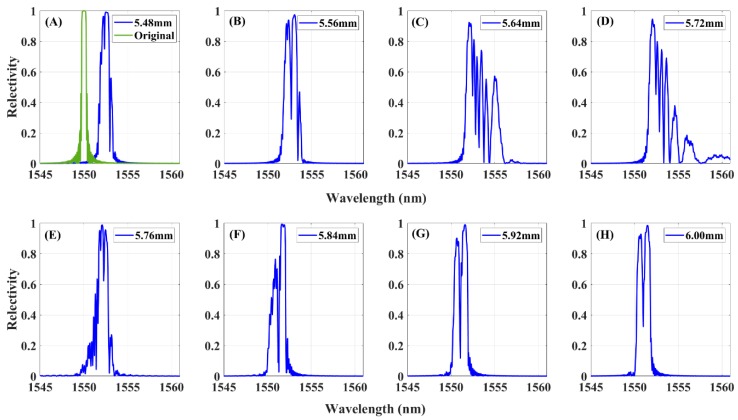
Simulated reflected spectra in subfigures (**A**–**H**) of the FBG1 sensor with applied strain field with the same crack lengths of Figure 2.

Figure 3 shows the simulated reflected spectrum of the FBG1 sensor subjected to the applied strain distribution of Figure 2. The reflected spectra are windowed to a wavelength range of 1545–1560 nm to present the wavelength shift of the peak. The original spectra with zero mean strain are simulated in green, with the crack propagation near the FBG1 sensor, and the reflected spectrum shifted toward to the long wavelength direction and was also distorted. Additionally, a significant decrease in reflection peak power is also observed with crack propagation close to the sensor. Other forms accompany the spectral distortion including spectral broadening and appearance of multiple peaks.

### 2.3. Damage Feature—FWHM

Previous studies have demonstrated that the spectral bandwidth is an indicator to measure the degree of FBG spectral distortion due to the localized resonances at different wavelengths created by an induced strain along the FBG [22]. Thus, the spectral bandwidth represents the magnitude of strain distribution ranges and the strain gradient value across the FBG. The simulated reflected spectra versus crack lengths are shown in Figure 3. The normalized reflected signal responses ≥0.5 were selected, and the wavelength range of these responses was defined as FWHM, the full width at half maximum.

**Figure 4:**
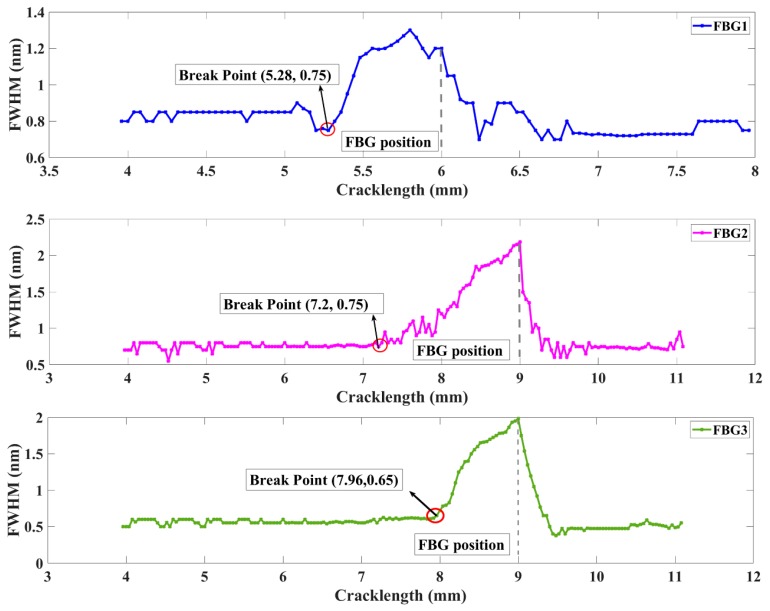
Calculated FWHM for simulated FBG responses under strain extracted by FEM method.

Figure 4 shows the spectral bandwidth extracted from a simulated FBG sensor response. As expected, the FWHM gently varies with the crack propagation and when it approximately contacts the sensors, there would be a sharp increase. In the initiation stage of crack growth, the stress concertation area is distant enough from the location of the FBGs, and the grating senses the uniform strain, which will obtain a single symmetric Gaussian peak and produce a central wavelength shifting. Next, with the crack propagation near the FBGs, the inhomogeneous plastic zone formed ahead of the crack tip will approximately cross the grating, creating spectral distortion and even a primary peak splitting, and the FWHM has an increasing trend. Additionally, the FBGs bonded perpendicular to the crack propagation path would sense the entire plastic zone developed at the crack tip and the inhomogeneous symmetrical strain with gradient. As the applied fatigue loading cycles increase, the plastic zone moves far away from the FBGs, and the elastically strained material behind the crack tip unloads. Thus, the shape of the FBGs reflected spectra trend toward recovery and the FWHM of the FBGs gradually decline. Moreover, it is shown that the fatigue crack damage localization corresponds to the saltation points in the FWHM curve, and the sensitivity distance to crack detection of FBG2 (1.8 mm) is longer than that of FBG1 (0.72 mm) and FBG3 (1.04 mm). This simulation data result shows that the FWHM is an excellent feature for crack location. The complicated experimental data test is discussed in Section 3.

### 2.4. Damage Feature—Spectral Difference

**Figure 5:**
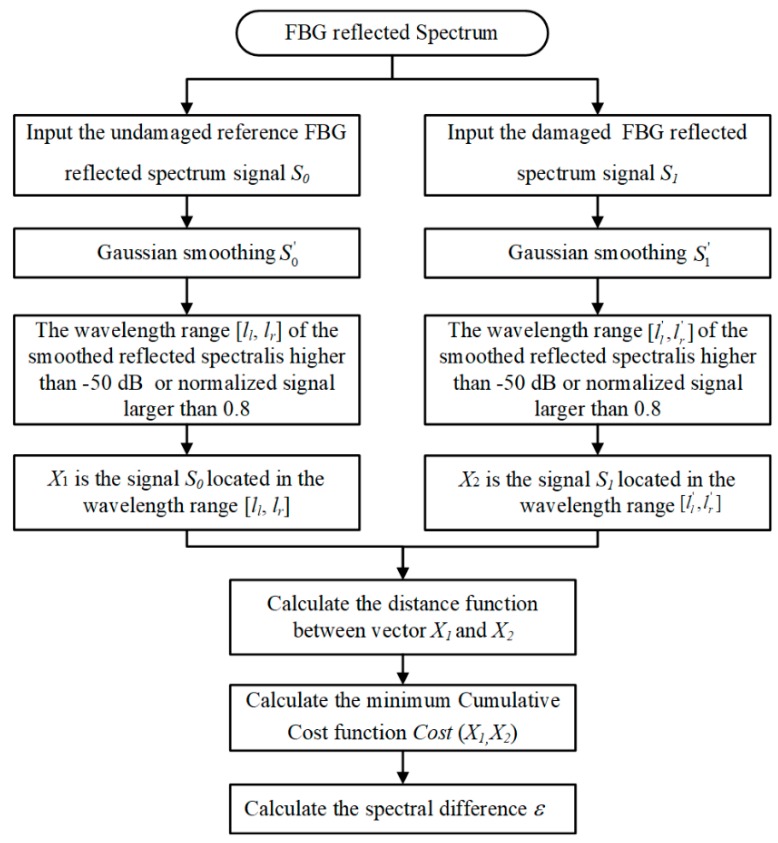
Flow of spectral difference based on the DTW method.

The second proposed damage parameter is the spectral difference based on the dynamic time warping (DTW) method [23]. It is a quantitative indicator to estimate the two series of FBG deformed reflected spectra and healthy reference spectrum for the same sensor. The damage feature is only sensitive to spectral broadening and spectral deformation and ignores the effect caused by central wavelength shifting. The proposed algorithm overcomes the traditional Euclidean-distance definition limitations that are overdependent on central wavelength shifting phenomenon and need to satisfy the same comparative signal lengths requirement. The spectral difference based on the DTW algorithm flow is shown in Figure 5.

The spectral difference based on the DTW method is defined as follows:

*Step 1*: The reference FBG reflected spectrum signal, which is received in undamaged material conditions, is written as S_0_, and the data were processed to Gaussian smoothing. The Gaussian convolution kernel was adapted with the length of 120, mean value of 0, and standard deviation of 20. The width [*l_l_*, *l_r_*] shows that the wavelength range of the smoothed reflected spectral $S_{0}^{\prime }$ is higher than −50 dB or normalized signal larger than 0.8. The boundary forms the division between the useful signal and noise data for each FBG sensor. *X*_1_ is the signal located in the wavelength range [*l_l_*, *l_r_*] and the length approximates to *N*.

*Step 2*: A similar approach to the real-time damage signal S_1_ and the smoothing data $S_{1}^{\prime }$ was utilized after the Gaussian smoothing process. The $\left[l_{l}^{\prime },l_{r}^{\prime }\right]$ is used to determine the corresponding wavelength of $S_{1}^{\prime }$ data which is larger than −50 dB or normalized signal larger than 0.8. The length of signal *X_2_* satisfies the wavelength range $\left[l_{l}^{\prime },l_{r}^{\prime }\right]$ and approximates to *M*.

*Step 3*: The smoothing spectrum can be expressed by a sequence of feature vectors.

(1) $$\begin{array}{l} X_{1}=X_{1,1},X_{1,2},X_{1,3},\ldots X_{1,n-2},X_{1,n-1},X_{1,n}\\ X_{2}=X_{2,1},X_{2,2},X_{2,3},\ldots X_{2,m-2},X_{2,m-1},X_{2,m} \end{array}$$

*Step 4*: To clarify the wavelength length differences between these two spectral patterns, an *n* − *m* plane is considered, for which the spectral patterns $X_{1}$ and $X_{2}$ are developed along the *n*-axis and the *m*-axis with the same category. The wavelength differences between patterns can be described by a series of points *c* = (*n*, *m*):

(2) $$\begin{array}{l} c(n,m)=dist(X_{1,n},X_{2,m}):\\ F=c(1),c(2),\ldots c(k),\ldots ,c(K), \end{array}$$

where

c(k)=(n(k),m(k)).

This sequence presents an approximate warping function from the wavelength axis of pattern $X_{1}$ to that of $X_{2}$. The warping function coincides with the diagonal line *n* = *m* when there is no wavelength difference between these patterns. However, as the wavelength difference grows, it deviates further from the diagonal line. As a distance is employed between the two feature vectors $X_{1,n}$ and $X_{2,m}$,

(3) $$dist(c)=dist(n,m)=\left\|X_{1,n}-X_{2,m}\right\|$$

The summation of the distances on weighted warping functions F becomes

(4) $$E(F)=\sum_{k=1}^{K}dist(c(k))\cdot w(k))$$

where a nonnegative weighting coefficient *w*(*k*) is introduced to keep the *E*(*F*) measurement flexible. The data point number on the warping function *F* is defined as *K*.

*Step 5*: By optimally adjusting the wavelength difference, the minimum value of the weighted warping function *F* is considered to be the spectral difference between spectral patterns $X_{1,n}$ and $X_{2,m}$ after eliminating the wavelength difference, which is presented as follows:

(5) $$Cost(X_{1},X_{2})=\underset{F}{Min}\frac{\sum_{k=1}^{k=K}dist(F_{k})}{\sum w(k)}$$

where the denominator ∑w(k) is introduced to compensate for the warping number effect.

Since the criterion function in (5) is a rational expression, its maximization is an unwieldy problem. If the denominator in (5)

(6) $$P\;=\;\overset{K}{\underset{k\;=\;1}{\sum }}w\left(k\right)$$

(Called normalization coefficient) is independent of warping function *F*, it can be simplified in the equation as follows:

(7) $$Cost\left(X_{1},X_{2}\right)\;=\;\frac{1}{P}\begin{array}{c} Min\\ F \end{array}\overset{k\;=\;K}{\underset{k\;=\;1}{\sum }}dist\left(F_{k}\right)$$

This simplified problem can be effectively solved by use of the dynamic programing technique and the typical weighting coefficient definitions enable this simplification. Additionally, it is knowing that in the FBG signal, the $\;Cost\left(X_{1},X_{2}\right)\;=\;Cost\left(X_{2},X_{1}\right)$, and it is assumed that was the symmetric form.

The weight coefficient definitions can be definition as follows.

(8) w(k) = (n(k)−n(k−1))+(m(k)−m(k−1))

Then

(9) N=N+M

where N and M are the lengths of smoothing FBG spectrum signal $X_{1}$ and $X_{2}$.

*Step 6*: The spectral difference ε is equal to the value of minimum cumulative cost function at the optimal weight warping.

**Figure 6:**
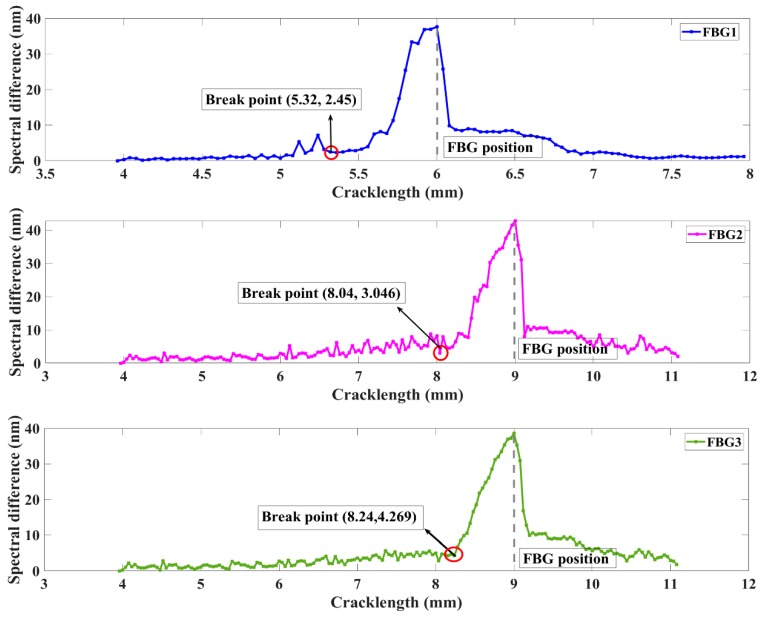
Spectral difference for simulated FBG responses under strain extracted by FEM method.

As expected, based on the simulated data, the calculated spectral difference varies with different crack lengths as shown in Figure 6. The spectral difference is a quantitative parameter to present the overlapping degree between measured distorted and healthy reflected spectra for the same sensor. This damage feature shows small fluctuations when the crack is at a far distance from the FBG sensor. Then, a monotonic and rapidly increase appears when the crack propagation distances to FBG1, FBG2, and FBG3 are within 0.68, 0.96, and 0.76 mm, respectively. When the crack grows cross through the sensors, due to the elastically strained material behind the crack tip unloads, the feature accompanying descends with the reflected spectra tending to return to its original state. Through the above analysis, the spectral difference is an excellent feature to estimate whether the crack is located in the monitoring area. Moreover, the 10 mm FBG shows a more border damage detection distance than the 5 mm FBG. The robustness of the feature to the experimental noise strain fields are evaluated in Section 3.

## 3. The Experimental Procedure

**Figure 7:**
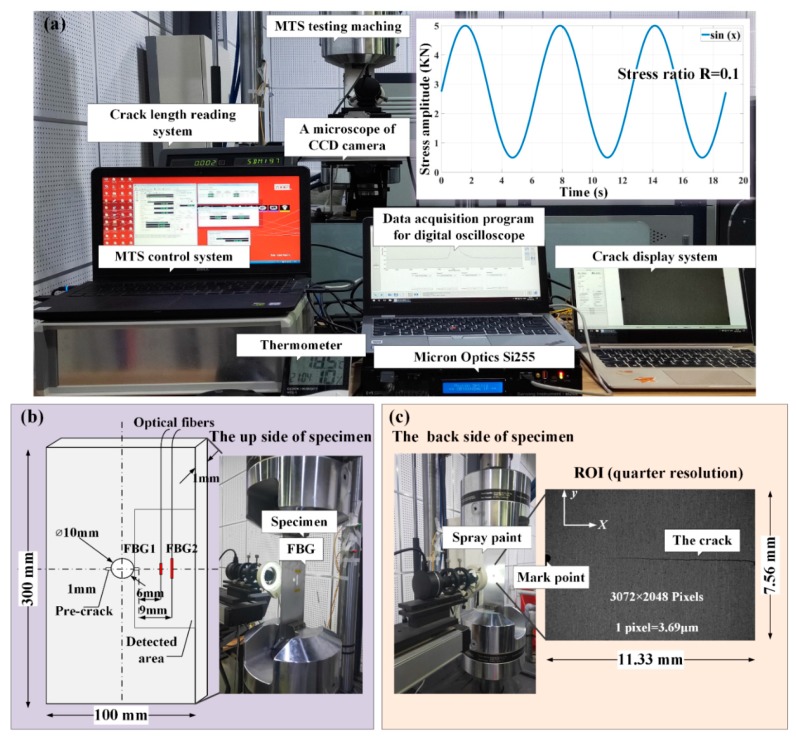
(**a**) A photo of the test setup; (**b**) experimental setup of fatigue crack monitoring using an FBG sensor; and (**c**) experimental setup of the DIC by CCD camera.

This study used a hole-edge crack damage detection fatigue experiment platform. The experiment platform contained a fatigue crack measurement system, an optical sensing system, a DIC measurement system and a fatigue load-cycling system, as shown in Figure 7a. To eliminate the temperature effect on the FBG reflected spectra, set the experiment temperature to 18.5 °C by a central air conditioning unit. Uniform tensile loading was applied at the bottom of the specimens, with the top boundary fixed. The load frequency of 15 Hz along the z-direction with a holding load was set to 40 MPa, the ratio was set to 0.1, and a sinusoidal wave fatigue load was undertaken with a maximum of 50 MPa by a hydraulic MTS machine, as shown in Figure 7a. Two parallel experiments were designed. On the upside of the specimens, the two FBG sensors were bonded perpendicular to the crack line on the top of a plate with 1 mm thickness epoxy resin adhesive and the gluing length is more than 2 times the FBG length. This setup served as the local-level sensor to sense axial strain variations with crack propagation, as shown in Figure 7b. Considering the reference region in DIC experiment, FBG1 should be far enough from the crack tip. Hence, a 6 mm distance was placed between the center hole and FBG1. A radial spacing of 3 mm of two sensors were located to evaluate the crack influence to the sensitivity damage detection of FBGs. The reflected spectrum response of each FBG sensor (mode number FSSR5025) was acquired after each millimeter of crack propagation within the range of 1–12 mm using the Micron Optics si255 interrogator. The wavelength measurement step size was 0.01 nm, which was equal to the previously obtained simulation accuracy. On the backside of two specimens, one is designed for the DIC experiment (in specimen 1), another is designed for the crack damage detection ability analysis of two proposed features (in specimen 2).

Therefore, a LEICA microsystem 8-bit intensity resolution CCD camera was used as an optical microscope and fixed to monitor the fatigue crack growth during the loading process. The strain field located perpendicular to the crack propagation direction at each millimeter step was collected and serviced as the current and reference image. In Figure 7c, the region of interest (ROI) (approximately 11.33 × 7.56 mm) includes the crack tip region. The resolution of the picture was achieved to 3.69 μm/pix. To realize the observation areas, the marked point was obtained within the ROI. The subset sizes and spacings of the DIC spatial resolution were 55 × 55 μm (15 × 15 pix) and 29.5 μm (8 pix), respectively. Additionally, in specimens2, a FBG3 sensor with grating lengths of 5 mm was designed at the backside of the plate. Moreover, the position of FBG3 is same to FBG2 and serviced as a comparison sensor.

## 4. Experimental Results and Discussion

### 4.1. Results—The Strain Detected by DIC with FBG Spectra Variation

**Figure 8:**
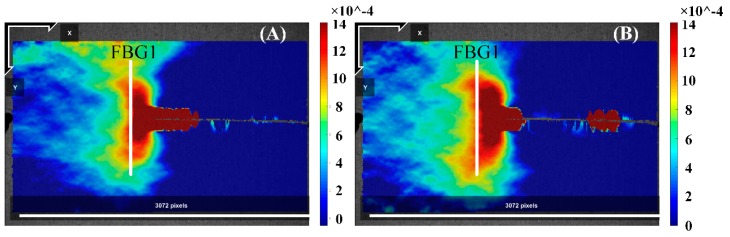
DIC plot of the strain fields around the FBG1 sensor in subfigures (**A**,**B**) crack lengths of 5.48 and 5.56 mm.

The DIC experimental measurements of the strain fields in the FBG sensor location were focused on the high-resolution strain fields at several crack growth lengths. To investigate the strain loading along the grating during a growing fatigue crack, the DIC method uses image processing techniques to obtain a one-to-one correspondence between small subsets in the initial unformed picture and subsequent deformed images. The respective locations and the strain information in the current configuration were obtained through a matching subset location transformation. Additionally, a spacing parameter (8 pix) was used to reduce the computational costs. In the end, a grid contained the strain information with respect to the reference configuration, also referred to as Lagrangian strains. The strain fields can then either be reduced or interpolated to form a continuous strain field. Then, the strain fields around the FBG1 sensor at crack lengths of 5.48 and 5.56 mm are shown in Figure 8. As a fatigue crack grows under different crack conditions, the extracted strain loading along the grating provided an insight into the stress concentration in the crack tip of the width-limited plate with holes with the crack propagation. Additionally, the results confirmed the finite-element results of the crack is introduced to the symmetrical nonuniformity strain field.

**Figure 9:**
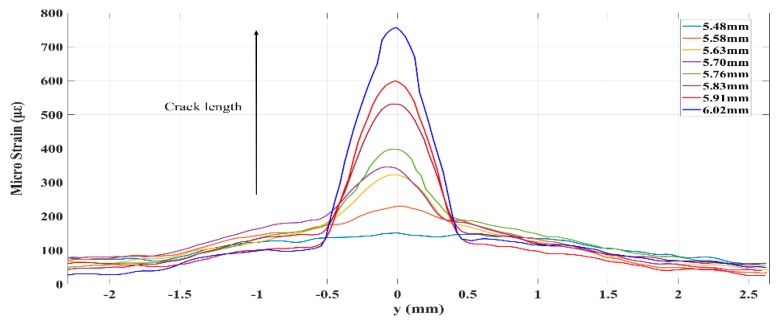
Strain along the grating of the FBG1 sensor at different crack lengths.

**Figure 10:**
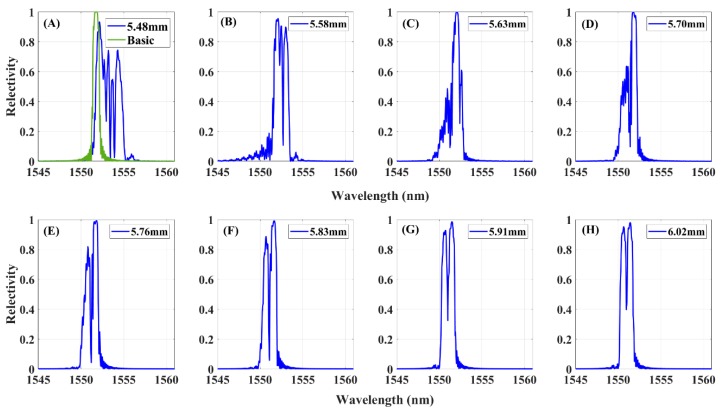
Experimental FBG1 reflected spectra in subfigures (**A**–**H**) at different crack lengths.

The DIC technology was analyzed in MATLAB environment, and the code was provided by Václav Nežerka [24]. In Figure 8, all DIC images (reference and conference) were processed to 3072 by 2048 square pixels, respectively. For example, there is an over plot of the strain field at the crack propagation site before the position of FBG1 was bonded. At crack length of 5.48 and 5.58 mm in Figure 9, the strain field of FBG1 near the crack tip is shown in Figure 8. Additionally, the DIC plot show the butterfly region at the crack tip. For a crack under monotonical load, the plastic zone from the crack tip consists of two high strain lobes emanating at some angle on each side of the crack propagation direction. Additionally, the experiment strain in Figure 9 contained similar values to the simulation results based on XFEM under similar crack lengths in Figure 2a. Then, the corresponding experimentally reflected spectra are shown in Figure 10.

The experimental data of FBG1 sensor, were subject to the fatigue loading impact. These reflected spectra were affected by multiple complex strain fields and noise disturbances. The reflected spectra of FBG1 are presented in Figure 10 at the same crack lengths in Figure 9, which shows a similar response to the modified-TMM simulation spectrum. The original reflected spectrum presented a narrow-bandwidth Gaussian envelope. When the crack propagation closed to FBG1, an inhomogeneous strain of the plane occurred, and the FBG1 sensor sensed the plastic zone ahead of the crack tip, for which the plasticity strain differs from the former plane elastic stress. As a result, the spectrum moved to a longer wavelength direction, the spectra distorted, presenting a broadened bandwidth, multiple peaks splitting from the main peak, and the appearance of a significant secondary peak at higher wavelengths. The measured data are used to evaluate the performance of the damage features for damage detection capability and noise robustness.

### 4.2. Results—Damage Location Measurement

The experimental reflected spectra are used to evaluate whether the proposed two damage parameters can detect crack damage even under the experimental complex strain field and noise interference. The different grating lengths sensors were located with different distances to the crack. Then, the signals of three FBGs were used to evaluate the crack detection capabilities and distinguish the influence of two differences (grating lengths and crack location).

**Figure 11:**
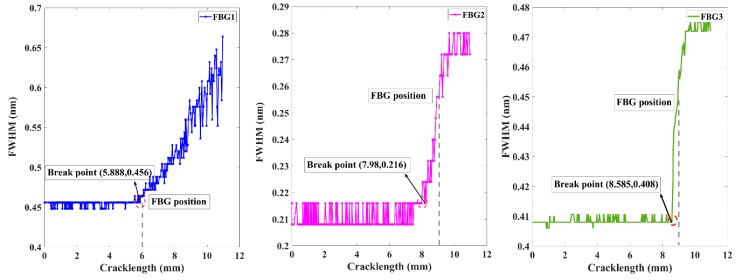
Experimental damage feature FWHM variation with different crack lengths.

The graphs of Figure 11 show the FWHM response of each sensor. The first step to calculate the measured FWHM were normalized by the reflectivity peak. Then, it can eliminate the effects of maximum measured reflectivity power losses in the optical fiber. The normalized signal data with ρ ≥ 0.5 were selected to investigate FWHM variations. As expected, the FWHM remained steady at the beginning of crack initiation. When the crack propagation is closer to the sensors, the FWHM increased monotonically with increasing damage accumulation. Additionally, the break point was a sensitive monitoring indicator which was associated with the moment of crack that arrived at the detection area. Moreover, the results were consistent with the simulation results discussed above. The damage feature FWHM showed a longer sensitivity detection distance of FBG2 (1.02 mm) compared to FBG1 (0.12 mm) and FBG3 (0.415), providing considerably long-distance monitoring advantages. When the FWHM of FBG1 changed, the FWHM of sensor FBG2 did not change simultaneously, demonstrating the robustness of the FWHM to experimental noise. The above research can provide support for further research. The difference between the simulated and experimental FWHMs of when the crack propagated through the sensors was primarily due to the tensile phenomenon of the grating fiber caused by crack opening displacement.

**Figure 12:**
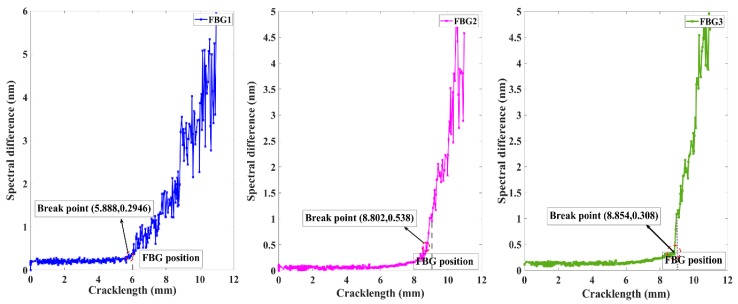
Experiment damage feature spectral difference variation with different crack lengths.

The behavior of the spectral difference for the two FBG sensors is presented in Figure 12. The feature performance shows a similar tendency to that of other FWHM measures. The FBG1 and FBG2 both demonstrated a steady trend until the crack propagated to the sensor, which was followed by a monotonic increase as damage accumulated when the crack grew near the FBG sensor. These differing behaviors of the break points presented the robustness of the experimental noise. This method overcomes the limitations of the traditional definition, which may be easily corrupted by environment noise, and the requirement of the same signal length. Additionally, the sensitivity distance of the spectral difference was longer in FBG2 (0.2 mm) than in FBG1 (0.12 mm) and FBG3 (0.146 mm). The difference between the simulated and experimental spectral difference after the crack propagated through the sensors was primarily due to the tensile phenomenon of the grating fiber caused by crack opening displacement and the complexity strain field.

In the practice, the proposed crack detection method based on damage features is important for the hole-crack detection monitoring and aircraft structural behavior evaluation. However, as the structural analysis becomes even more complex, it may consider the loads factors, materials factors, human factors, and the exposed environmental factors of structures. In order to eliminate the interference of above factors, the different types of sensors are distributed near the FBG crack sensors under a free-strain condition and applied as comparison sensors, such as the FBG temperature sensor and FBG curvature sensor. Then, a typical health monitoring system is composed by a network of sensors that measure a series of parameters relative to the structure and to its environment. What is more, is the ground FBG signal database that will bring a deeper knowledge of how this structure behaves. Additionally, the crack damage procedure is obtained by analyzing the features difference of real-time signal and the healthy basic signal. The stability of the methods has been tested under the full-scale fatigue and residual strength test of XX-aircraft.

## 5. Conclusions

The crack damage location detection was carried out using FBG sensors by analyzing the changing of damage features. Compared to the other research, the advantage of this technique was concluded in the following four aspects. Firstly, the study highlighted the reflected spectrum deformation mechanism with crack propagation by analyzing strain field variation along grating based on both the simulation and experiment method. The simulated FBG reflected spectra were measured by the modified-TMM method applied with strain states, which were extracted from FEM. The experimental strain along the grating was obtained by DIC technology, which is a kind of noncontact measurement method. In this connection, a useful viewpoint was introduced to explain the relationship between the FBG spectral deformation level and the strain state caused by crack propagation. Secondly, the performance of two potential damage features—FWHM and the spectral difference were evaluated. At the same time, the performance of two damage features were compared under simulations and experiments environment. The experimental results indicated that the two damage features excellently indicate the presence of damage with the characteristics that insensitivity and strong robustness to noise. Both damage features were sensitive to the spectral deformation due to the strain gradient and complex strain profile along the FBG axis caused by crack damage. The application of two sensitivity features would provide a robust measure of damage location within a material. Thirdly, the experimental trends of the two damage features from the bonded FBG sensors presents a significant break point corresponding to the moment of crack propagation toward to the damage detection region. The break point phenomenon was due to crack variations in the surrounding material that affect the damage accumulation loading near the FBG. Such models provide simple measures to detect the crack location according to these feature responses. At the end of this paper, due to the variability in response between the two different grating lengths sensors employed at the same damage conditions, the detection sensitivity results of the FBG sensors under different damage states show that a 10 mm FBG sensor may show superior monitoring distance performance compared to a 5 mm FBG sensor for crack position detection. In the future, the stability of the methods will be tested under a real experiment, such as the full-scale fatigue and residual strength test of XX-aircraft.

## Figures and Tables

**Table 1:** The information of FBG sensors.

Sensor Number	The Mechanical Properties of FBG Sensors					The Distance between FBGs and Original Crack-Tip (mm)	The Sensors on which Side of Specimen
	Grating Length (m)	Effective Index	Bragg Wavelength (nm)	Poisson’s Ratio (MPa)	Average Index Change		
FBG1	0.0051	1.450	1550	0.17	2.0 × 10^−4^	6	upside
FBG2	0.0100	1.458	1550	0.17	2.6 × 10^−4^	9	upside
FBG3	0.0051	1.450	1550	0.17	2.0 × 10^−4^	9	backside
